# UVRAG Deficiency Exacerbates Doxorubicin-Induced Cardiotoxicity

**DOI:** 10.1038/srep43251

**Published:** 2017-02-22

**Authors:** Lin An, Xiao-wen Hu, Shasha Zhang, Xiaowen Hu, Zongpei Song, Amber Naz, Zhenguo Zi, Jian Wu, Can Li, Yunzeng Zou, Lin He, Hongxin Zhu

**Affiliations:** 1 Bio-X-Renji Hospital Research Center, Renji Hospital, School of Medicine, Shanghai Jiao Tong University, Shanghai, China; 2 Institutes of Biomedical Sciences, Fudan University, Shanghai, China; 3 Collaborative Innovation Center of Genetics and Development, China

## Abstract

Doxorubicin (DOX) is an effective chemotherapeutic drug in the treatment of various types of cancers. However, its clinical application has been largely limited by potential development of cardiotoxicity. Previously we have shown that ultra-violet radiation resistance-associated gene (UVRAG), an autophagy-related protein, is essential for the maintenance of autophagic flux in the heart under physiological conditions. Here, we sought to determine the role of UVRAG-mediated autophagy in DOX-induced cardiotoxicity. Mouse models of acute or chronic DOX-induced cardiotoxicity were established. UVRAG deficiency exacerbated DOX-induced mortality and cardiotoxicity manifested by increased cytoplasmic vacuolization, enhanced collagen accumulation, elevated serum activities of lactate dehydrogenase and myocardial muscle creatine kinase, higher ROS levels, aggravated apoptosis and more depressed cardiac function. Autophagic flux was impaired in DOX-induced cardiotoxicity. UVRAG deficiency aggravated impaired autophagic flux in DOX-induced cardiotoxicity. Intermittent fasting restored autophagy and ameliorated pathological alterations of DOX-induced cardiotoxicity. Collectively, our data suggest that UVRAG deficiency exacerbates DOX-induced cardiotoxicity, at least in part, through aggravation of DOX-induced impaired autophagic flux. Intermittent fasting, which restores blunted autophagic flux and ameliorates pathology in the mouse models of DOX-induced cardiotoxicity, may be used as a potential preventive or therapeutic approach for DOX cardiotoxicity.

Doxorubicin (DOX) is an effective chemotherapeutic agent in the treatment of a broad range of human solid and hematogenous malignancies. However, clinical application of DOX is largely limited by its cardiotoxicity^1^,^2^. DOX-induced cardiotoxicity can be acute and chronic. Acute DOX cardiotoxicity occurs during 2–3 days of its administration, while chronic DOX cardiotoxicity is evident several weeks or even years after its administration. The incidence of DOX cardiotoxicity is related to its cumulative dose^3^. However, the precise mechanisms of DOX-induced cardiotoxicity have not been completely understood. Reactive oxygen species (ROS) is widely accepted as the major mediator of DOX-induced cardiac injury^4^,^5^. In addition, ubiquitinated-protein aggregation, apoptotic cell death, cytoplasmic vacuolization, collagen deposition of myocardial cells, inflammation and iron accumulation in the mitochondria also contribute^5^,^6^,^7^.

Macroautophagy (hereafter referred to as autophagy) is an evolutionarily conserved process delivering cytoplasmic components to lysosome for degradation and recycling. Autophagy process in mammalian cells consists of several sequential stages-autophagosome formation, maturation, lysosomal degradation and recycling of degradation products. Among them, autophagosome formation can be further divided into four steps: initiation, nucleation, elongation and closure^8^. Modulation of any step of autophagy process can alter autophagic flux. In the heart, autophagy occurs in the basal states, which is essential to maintain cellular homeostasis^9^,^10^. Deregulation of cardiac autophagy is associated with a variety of heart diseases^11^,^12^,^13^. Autophagy has recently been linked to DOX-induced cardiomyopathy^14^,^15^,^16^,^17^,^18^,^19^. However, whether cardiac autophagy is activated or impaired and which specific step is modulated in DOX-induced carditoxicity is not completely understood. In addition, the functional significance of deregulated autophagy in DOX-induced cardiotoxicity is still under debate.

Ultraviolet irradiation resistance-associated gene (UVRAG), an autophagy-related protein, has been shown to regulate autophagosome formation^20^, maturation^21^ and autophagic lysosomal reformation (ALR)^22^. We have recently obtained a line of UVRAG-deficient mice screened by piggyBac transposition and demonstrated that UVRAG deficiency leads to impaired autophagic flux accompanied with accumulated autophagosomes in the heart, suggesting that UVRAG regulates autophagosome maturation of cardiac autophagy^23^,^24^. In the present study, we sought to determine the role of UVRAG-mediated autophagy in DOX-induced cardiotoxicity. Using UVRAG-deficient mice combining experimental mouse models of acute and chronic DOX-induced cardiotoxicity mimicking those in patients, we show for the first time that UVARG deficiency exacerbates DOX-induced cardiotoxicity. In addition, UVRAG deficiency aggravates DOX-induced impaired autophagic flux in the heart. Finally, we provide evidence that intermittent fasting rescues impaired autophagy and ameliorates pathological alterations in DOX cardiotoxicity.

## Results

### UVRAG deficiency exacerbates acute DOX-induced cardiotoxicity

**Figure 1:**
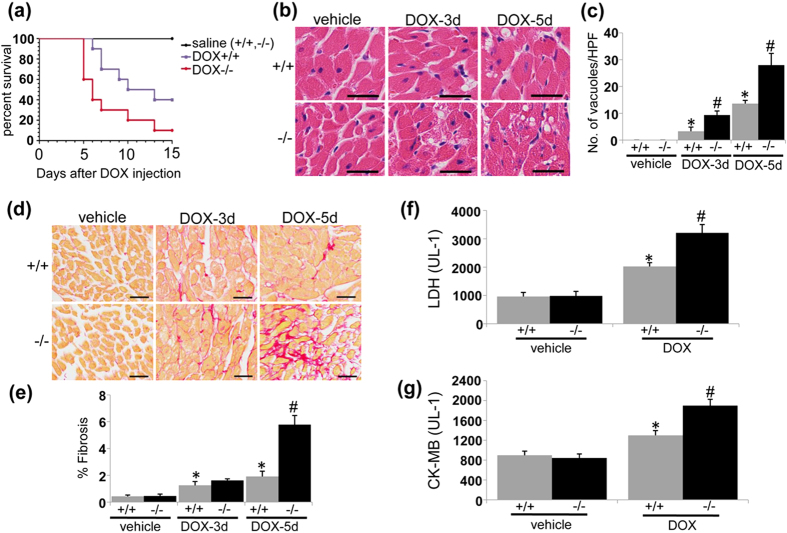
UVRAG deficiency exacerbates acute DOX-induced cardiotoxicity. (**a**) Survival curves of WT and UVRAG-deficient mice after acute DOX treatment were created by Kaplan-Meier method (*P < 0.05 using the Log-rank test). n = 7 mice for saline+/+, n = 8 mice for saline−/−, n = 10 mice for DOX+/+, n = 10 mice for DOX−/−. (**b**) Representative H&E images of degenerative vacuoles in LVs on heart sections from WT and UVRAG-deficient mice 3 or 5 days after acute DOX or vehicle treatment. Scale bar: 40 μm. (**c**) Quantification of degenerative vacuoles in LVs in the experiments as illustrated in (**b**). n = 3 mice for each group. (**d**) Representative images of fibrosis stained with picrosirius red in LVs on heart sections from WT and UVRAG-deficient mice 3 or 5 days after acute DOX or vehicle treatment. Scale bar: 40 μm. (**e**) Quantification of fibrosis in LVs in the experiments as illustrated in (**d**). n = 3 mice for each group. (**f**) Serum LDH activity in WT and UVRAG-deficient mice 3 days after acute DOX or vehicle treatment. n = 4 mice for each group. (**g**) Serum CK-MB activity in WT and UVRAG-deficient mice 3 days after acute DOX or vehicle treatment. n = 4 mice for each group. *P < 0.05 vs. WT + vehicle, ^#^*P* < 0.05 vs. WT + DOX.

Young adult UVRAG-deficient mice we obtained have normal cardiac morphology and function^23^. UVRAG protein was not detected in the hearts from UVRAG-deficient mice after vehicle or DOX treatment (Fig. S1). In acute DOX-induced cardiotoxicity, the survival rate of DOX-treated UVRAG-deficient mice was significantly lowered compared with DOX-treated wild type (WT) control mice (Fig. 1a). We then examined cytoplasmic vacuolization, a hallmark of cardiomyocyte degeneration^5^, in left ventricles (LVs) in DOX-induced cardiotoxicity. We barely detected vacuolar degeneration of cardiomyocytes from vehicle-treated WT and UVRAG-deficient mice. As expected, DOX-treated WT mice showed significant increase in degenerative vacuoles (Fig. 1b,c). Importantly, UVRAG-deficient mice showed more degenerative vacuoles compared with WT controls after DOX treatment (Fig. 1b,c). No fibrosis was detected in vehicle-treated WT and UVRAG-deficient mice. After DOX treatment, fibrosis was present in WT and UVRAG-deficient mice (Fig. 1d). However, UVRAG-deficient mice developed more pronounced fibrosis 5 days after DOX treatment (Fig. 1d,e). In agreement with these findings, the expression of α-SMA, a marker of active cardiac fibroblast^25^, was enhanced in LVs from DOX-treated UVRAG-deficient mice compared with WT controls (Fig. S2a,b). Moreover, the serum activities of lactate dehydrogenase (LDH) and myocardial muscle creatine kinase (CK-MB), two important biomarkers for cardiac injury^26^, rose significantly in DOX-treated UVRAG-deficient mice compared with DOX-treated WT controls (Fig. 1f,g). Taking together, these data demonstrate that UVRAG deficiency exacerbates acute DOX-induced cardiotoxicity.

### UVRAG deficiency exacerbates chronic DOX-induced cardiotoxicity

**Figure 2:**
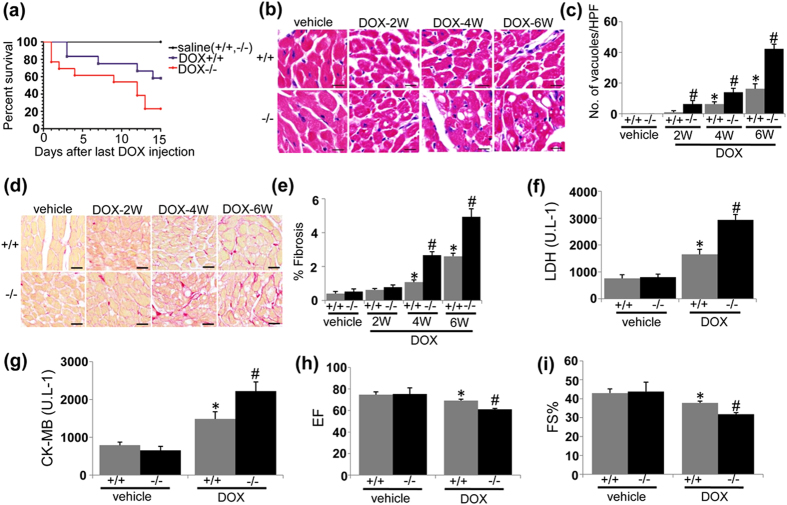
UVRAG deficiency exacerbates chronic DOX-induced cardiotoxicity. (**a**) Survival curves of WT and UVRAG-deficient mice after chronic DOX treatment were created by Kaplan-Meier method (*P < 0.05 using the Log-rank test). n = 6 mice for saline + / + , n = 6 mice for saline−/−, n = 12 mice for DOX + / + , n = 13 mice for DOX−/−. (**b**) Representative H&E images of degenerative vacuoles in LVs on heart sections from WT and UVRAG-deficient mice after chronic DOX or vehicle treatment over the indicated time. Scale bar: 40 μm. (**c)** Quantification of degenerative vacuoles in LVs in the experiments as illustrated in (**b**). n = 3–5 mice for each group. (**d**) Representative images of fibrosis stained with picrosirius red in LVs on heart sections from WT and UVRAG-deficient mice after chronic DOX or vehicle treatment over the indicated time. Scale bar: 40 μm. (**e**) Quantification of fibrosis in LVs in the experiments as illustrated in (**d**). n = 3–5 mice for each group. (**f**) Serum LDH activity in WT and UVRAG-deficient mice 2 weeks after chronic DOX or vehicle treatment. n = 4 mice for each group. (**g**) Serum CK-MB activity in WT and UVRAG-deficient mice 2 weeks after chronic DOX or vehicle treatment. n = 4 mice for each group. (**h**) Left ventricular EF of WT and UVRAG-deficient mice after chronic DOX or vehicle treatment. n = 4–5 mice for each group. (**i**) Left ventricular FS of WT and UVRAG-deficient mice after chronic DOX or vehicle treatment. n = 4–5 mice for each group. *P < 0.05 vs. WT + vehicle, ^#^*P* < 0.05 vs. WT + DOX.

Next we examined the effects of UVRAG deficiency on chronic DOX-induced cardiotoxicity. Again, the survival rate of DOX-treated UVRAG-deficient mice was significantly reduced compared with DOX-treated WT controls (Fig. 2a). Cytoplasmic vacuolization was more apparent in UVRAG-deficient mice compared with WT controls at 4 and 6 weeks of DOX treatment (Fig. 2b,c). In addition, UVRAG-deficient mice showed more fibrosis at 4 and 6 weeks of DOX treatment (Fig. 2d,e). Consistently, α-SMA was upregulated in DOX-treated UVRAG-deficient mice compared with WT controls (Fig. S3a,b). Again, serum LDH and CK-MB activities were significantly higher in DOX-treated UVRAG-deficient mice compared with DOX-treated WT controls (Fig. 2f,g). Left ventricular ejection fraction (EF) and fractional shortening (FS%), reduced in DOX-treated WT mice, were further decreased in DOX-treated UVRAG-deficient mice, suggestive of more depressed cardiac function in UVRAG-deficient mice after chronic DOX treatment (Fig. 2h,i). These findings demonstrate that UVRAG deficiency exacerbates pathology of chronic DOX-induced cardiotoxicity.

### UVRAG deficiency increases ROS production in DOX-induced cardiotoxicity

**Figure 3:**
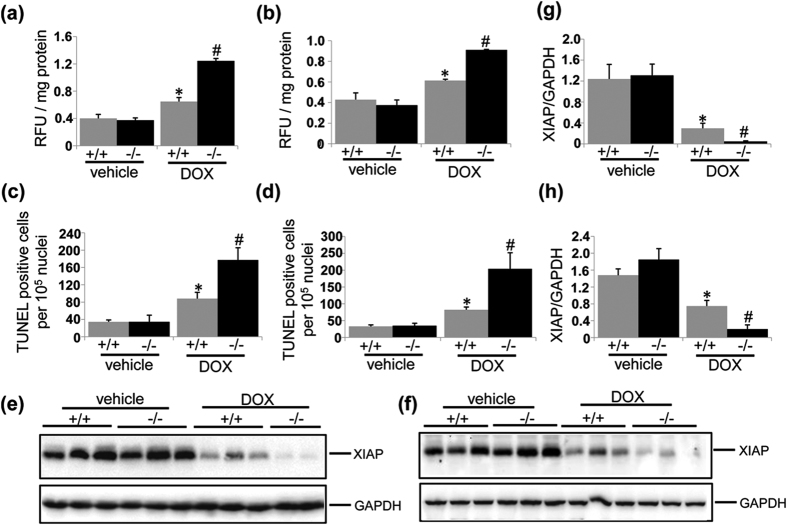
UVRAG deficiency increases ROS production and apoptosis in DOX-induced cardiotoxicity. (**a**) ROS levels in LVs detected using DCFH-DA 5 days after acute DOX or vehicle treatment. n = 3 mice for each group. (**b**) ROS levels in LVs detected using DCFH-DA at 4 weeks of DOX or vehicle treatment in chronic DOX cardiotoxicity. n = 3 mice for each group. (**c**) Quantification of apoptotic cells 5 days after acute DOX or vehicle treatment. n = 3 mice for each group. (**d**) Quantification of apoptotic cells at 4 weeks of DOX or vehicle treatment. (**e**) Western blot analysis of XIAP in LVs from WT and UVRAG-deficient mice 3 days after acute DOX or vehicle treatment. (**f**) Quantification of XIAP as illustrated in panel (e). (**g**) Western blot analysis of XIAP in LVs from WT and UVRAG-deficient mice at 4 weeks of chronic DOX treatment or vehicle treatment. (**h**) Quantification of XIAP as illustrated in panel (g). n = 3 mice for each group. *P < 0.05 vs. WT + vehicle, ^#^*P* < 0.05 vs. WT + DOX.

It is well established that DOX acts as an electron acceptor to stimulate mitochondria to generate ROS^27^, which has long been considered as the major mediator of DOX-induced cardiotoxicity^2^,^6^. Therefore, we assessed ROS levels using ROS-sensitive fluorescence probe DCFH-DA in acute and chronic DOX-induced cardiotoxicity. Given that LVs from DOX-treated UVRAG-deficient mice showed more dramatic difference in degenerative vacuoles and fibrosis 5 days after DOX treatment compared with DOX-treated WT controls, we chose 5 days as a time-interval to detect ROS production in acute cardiotoxicity. DOX treatment enhanced ROS levels in LVs from WT mice (Fig. 3a,b). In UVRAG-deficient mice, ROS levels were further enhanced after acute and chronic DOX treatment (Fig. 3a,b). These data suggest that exacerbation of DOX-induced cardiotoxicity by UVRAG deficiency is at least partially attributed to enhanced ROS levels.

### UVRAG deficiency enhances apoptosis in DOX-induced cardiotoxicity

Apoptosis, which can be triggered d by ROS, also contributes to DOX-induced cardiomyopathy^28^,^29^. First we performed TUNEL assays to detect apoptotic cell death in acute and chronic DOX-induced cardiotoxicity. TUNEL-positive cells were significantly increased in the hearts from WT mice after DOX treatment (Figs S4 and 3c,d). More importantly, the rate of TUNEL-positive cells was significantly higher in the hearts from DOX-treated UVRAG-deficient mice compared with DOX-treated WT controls (Figs S4 and 3c,d), indicating UVRAG deficiency enhanced apoptotic cell death after DOX treatment. We then performed western blot to detect X-linked inhibitor of apoptosis protein (XIAP), the most potent inhibitor of caspase 3 activity in IAP family^30^, to determine apoptosis. XIAP protein abundance was significantly reduced in the hearts from DOX-treated WT mice compared with vehicle-treated controls (Fig. 3e–h), suggestive of increased apoptotic cell after DOX treatment. Moreover, DOX-treated UVRAG-deficient mice showed significantly lower XIAP protein levels in the hearts compared with DOX-treated WT controls, suggesting UVRAG deficiency enhanced apoptotic cell death compared with WT controls after DOX treatment (Fig. 3e–h). Thus, these results suggest that increased apoptosis contributes to exacerbation of DOX-induced cardiotoxicity by UVRAG deficiency.

### UVRAG deficiency elevates inflammatory cytokines in the heart in DOX-induced cardiotoxicity

Growing evidence has shown that inflammation is induced in DOX-induced cardiotoxicity^31^. Therefore, we assessed inflammatory cytokines in the heart using ELISA method. TNFα (Fig. S5a,b), IL-6 (Fig. S5c,d) and IL-1β (Fig. S5e,f) levels in the heart rose significantly in DOX-treated mice compared with vehicle-treated controls. DOX-treated UVRAG-deficient mice showed elevated levels of TNFα, IL-6 and IL-1β in the hearts compared with DOX-treated WT control mice (Fig. S5a–f). Therefore, UVRAG deficiency promotes inflammation in DOX-induced cardiotoxicity.

### Autophagic flux is impaired in DOX-induced cardiotoxicity

**Figure 4:**
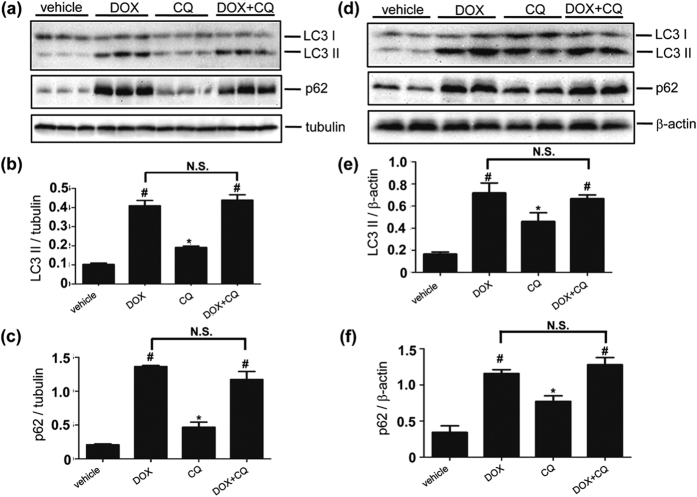
Autophagic flux is impaired in acute and chronic DOX-induced cardiotoxicity. (**a**) Western blot detection of LC3 II and p62 protein abundance in LVs from mice 5 days after acute DOX treatment and further elicited by CQ treatment. (**b**) Quantification of LC3-II protein abundance as illustrated in panel (a). n = 3 mice for each group. (**c**) Quantification of p62 protein abundance as illustrated in panel (a). n = 3 mice for each group. (**d**) Western blot detection of LC3 II and p62 protein abundance in LVs from mice treated with DOX over 4 weeks and further elicited by CQ treatment. (**e**) Quantification of LC3-II as illustrated in panel (d). n = 3 mice for each group. (**f**) Quantification of p62 as illustrated in panel (d). n = 3 mice for each group. *P < 0.05 vs. vehicle. ^#^*P* < 0.05 vs. CQ.

The ROS production contributes to mitochondrial damage, which in turn, stimulates more ROS production, forming a vicious cycle. In addition, damaged mitochondria are capable of triggering cell death^32^. Autophagy plays a housekeeping role in clearing damaged mitochondria in the heart to regulate mitochondria quality control, which reduces ROS generation and apoptosis^33^,^34^. We hypothesize that DOX impairs cardiac autophagy in DOX-induced cardiotoxicity, which is exacerbated by UVRAG deficiency due to aggravated impairment of autophagic flux. To test this hypothesis, we first examined autophagic flux in LVs from DOX-treated WT mice. In acute DOX-induced cardiotoxicity, we assessed autophagic flux 5 days after DOX treatment. LC3 II protein levels were significantly increased in DOX-treated mice compared with vehicle-treated controls. However, the accumulation of LC3 II in DOX-treated mice was not further increased by CQ treatment, suggesting attenuation of autophagic flux by DOX treatment (Fig. 4a,b). Interestingly, LVs from DOX-treated mice showed more pronounced LC3 II levels compared with CQ-treated mice (Fig. 4a,b). Moreover, p62 protein levels were markedly increased in DOX-treated mice. However, p62 accumulation in DOX-treated animals was not increased by CQ treatment, confirming impairment of autophagic flux after DOX treatment (Fig. 4a,c). In chronic DOX-induced cardiotoxicity, we measured autophagic flux at 4 weeks of DOX treatment. LC3 II and p62 protein abundance, upregulated after DOX treatment, were not further enhanced by CQ treatment (Fig. 4d–f). Again, LVs from DOX-treated mice showed more abundant LC3 II levels compared with CQ-treated mice (Fig. 4d,e). These data suggest that autophagic flux in the heart is blunted in DOX-induced cardiotoxicity.

### UVRAG deficiency aggravates impaired autophagic flux in DOX-induced cardiotoxicity

**Figure 5:**
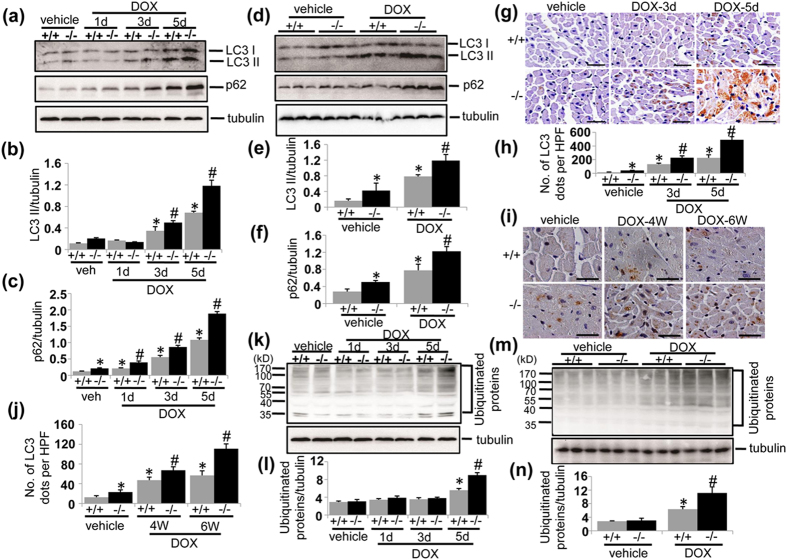
UVRAG deficiency aggravates impaired autophagy in DOX-induced cardiotoxicity. (**a**) Western blot detection of LC3 II and p62 protein levels in LVs from WT and UVRAG-deficient mice at the time points as indicated in acute DOX-treated mice. (**b**) Quantification of LC3 II protein abundance in the experiments as illustrated in (**a**). n = 3–5 mice for each group. (**c**) Quantification of p62 protein abundance in the experiments as illustrated in (**a**). n = 3–5 mice for each group. (**d**) Western blot detection of LC3 II and p62 protein levels in LVs from WT and UVRAG-deficient mice at 4 weeks of DOX or vehicle treatment in chronic DOX-treated mice. (**e**) Quantification of LC3 II protein abundance in the experiments as illustrated in (**d**). n = 3 mice for each group. (**f**) Quantification of p62 protein abundance in the experiments as illustrated in (**d**). n = 3 mice for each group. (**g**) Representative images of LC3 immunohistochemistry in LVs on heart sections from WT and UVRAG-deficient mice after acute DOX or vehicle treatment over the indicated time. Scale bar: 40 μm. (**h**) Quantification of LC3-positive dots in LVs in the experiments as illustrated in (**g**). n = 3 mice for each group. (**i**) Representative images of LC3 immunohistochemistry in LVs on heart sections from WT and UVRAG-deficient mice after chronic DOX or vehicle treatment over the indicated time. Scale bar: 40 μm. (**j**) Quantification of LC3-positive dots in LVs in the experiments as illustrated in (**i**). n = 3 mice for each group. (**k**) Western blot detection of ubiquitinated proteins in LVs from WT and UVRAG-deficient mice at the time points as indicated in acute DOX-treated mice. (**l**) Quantification of protein ubiquitination in the experiments as illustrated in (**k**). n = 3 mice for each group. (**m**) Western blot detection of ubiquitinated proteins in LVs from WT and UVRAG-deficient mice at the time points as indicated in chronic DOX-treated mice. (**n**) Quantification of protein ubiquitination in the experiments as illustrated in (**m**). n = 3 mice for each group. *P < 0.05 vs. WT + vehicle. ^#^*P* < 0.05 vs. WT + DOX.

**Figure 6:**
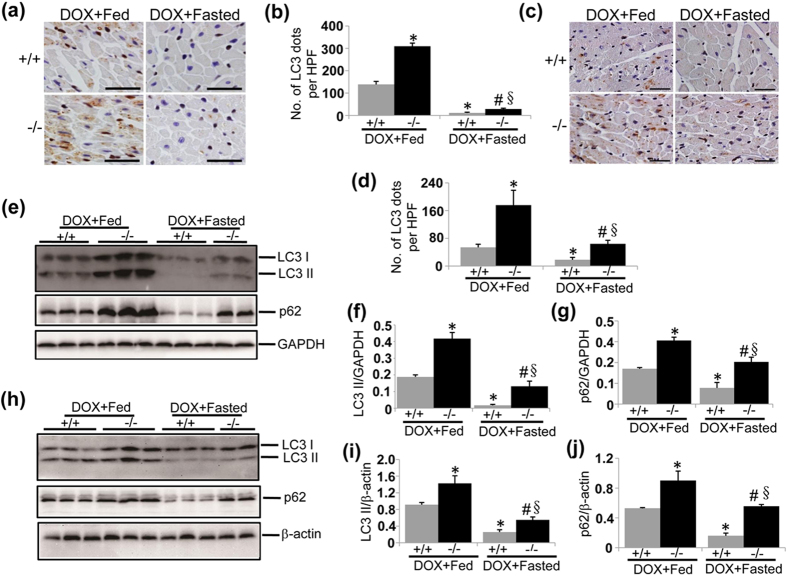
Intermittent fasting restores autophagy in DOX-induced cardiotoxicity. (**a**) Representative images of LC3 immunohistochemistry in LVs on heart sections from fed or fasted WT and UVRAG-deficient mice 5 days after acute DOX or vehicle treatment. Scale bar: 40 μm. (**b**) Quantification of LC3-positive dots in LVs in the experiments as illustrated in (**a**). n = 3 mice for each group. (**c**) Representative images of LC3 immunohistochemistry in LVs on heart sections from fasted WT and UVRAG-deficient mice at 4 weeks of DOX treatment in chronic cardiotoxicity. Scale bar: 40 μm. (**d**) Quantification of LC3-positive dots in LVs in the experiments as illustrated in (**c**). n = 3 mice for each group. (**e**) Western blot detection of LC3 and p62 in LVs from fed or fasted WT and UVRAG-deficient mice 5 days after acute DOX or vehicle treatment. (**f**) Quantification of LC3 II protein abundance in the experiments as illustrated in (**e**). n = 3 mice for each group. (**g**) Quantification of p62 protein abundance in the experiments as illustrated in (**e**). n = 3 mice for each group. (**h**) Western blot analysis of LC3 II and p62 in LVs from fed or fasted WT and UVRAG-deficient mice at 4 weeks of DOX treatment in chronic cardiotoxicity. (**i**) Quantification of LC3 II protein abundance in the experiments as illustrated in (**h**). n = 3 mice for each group. (**j**) Quantification of p62 protein abundance in the experiments as illustrated in (**h**). n = 3 mice for each gorup. *P < 0.05 vs. WT + DOX + Fed, ^#^*P* < 0.05 vs. UVRAG^−/−^ + DOX + Fed, ^§^*P* < 0.05 vs. WT + DOX + Fasted.

Next we examined whether UVRAG deficiency affected autophagic flux in DOX-induced cardiotoxicity. LC3 II and p62 protein abundance were significantly increased in LVs from UVRAG-deficient mice compared with corresponding WT controls in acute DOX cardiotoxicity (Fig. 5a–c) and at 4 weeks of DOX treatment in chronic DOX cardiotoxicity (Fig. 5d–f), indicating that UVRAG deficiency and DOX treatment showed an additive effect on cardiac autophagic flux. Consistently, immunohistochemistry showed more LC3-positive dots in LVs from UVRAG-deficient mice compared with WT controls after acute (Fig. 5g,h) and chronic (Fig. 5i,j) DOX treatment. Moreover, increased accumulation of ubiquitinated protein aggregates, a substrate for cardiac autophagy^35^, was induced in LVs from UVRAG-deficient mice 5 days after DOX treatment in acute DOX-induced cardiotoxicity (Fig. 5k,l) and at 4 weeks of DOX treatment in chronic DOX-induced cardiotoxicity (Fig. 5m,n). We also detected autophagy-related protein Beclin 1 and Atg5. Beclin 1 is involved in autophagy induction, while Atg5 forms a complex with conjugation to Atg12, which is essential for autophagosome formation^36^. As reported previously^23^, Beclin 1 protein levels were lower in the hearts from UVRAG-deficient mice compared with WT controls under normal conditions (Fig. S6a,e). Both acute and chronic DOX treatment increased Beclin 1 protein levels in WT and UVRAG-deficient mice compared with vehicle-treated controls (Fig. S6a,b,e,f), consistent with previous studies showing that DOX treatment induces autophagy induction. However, Beclin 1 protein levels were significantly lower in UVRAG-deficient mice compared with WT controls after acute DOX treatment (Fig. S6a,b). Interestingly, Beclin 1 protein levels in UVRAG-deficient mice were comparable to WT controls after chronic DOX treatment (Fig. 6e,f). Atg5 protein abundance remained unaltered in response to DOX treatment (Fig. S6c,d,g,h). Overall, these data suggest that UVRAG deficiency further impaired autophagic flux in DOX-induced cardiotoxicity.

### Intermittent fasting restores autophagy in DOX-induced cardiotoxicity

Short-term fasting restores autophagy in several pathological conditions^37^,^38^,^39^,^40^. However, autophagy returns to basal levels after short-term fasting is completed, which does not allow determining long-term effects of fasting-induced autophagy on DOX cardiotoxicity especially in chronic mouse models. It should be pointed out that prolonged fasting generates a variety of negative effects on the health. Therefore, we applied intermittent fasting, which avoids adverse effects produced by prolonged fasting and meanwhile boosts cardiac autophagy in a cyclic manner^41^, to the experimental mice. We sought to determine whether intermittent fasting is capable of rescuing autophagy in DOX-induced cardiotoxicity. To achieve this, we subjected WT and UVRAG-deficient mice to every-other-day fasting, starting one day before the initial injection of DOX. Again, we chose five days as a time-interval to determine the effects of intermittent fasting on acute cardiotoxicity. Upon acute DOX treatment, LC3-postive dots measured by immunohistochemistry were more pronounced in LVs from UVRAG-deficient mice compared with WT controls (Fig. 6a,b). Importantly, intermittent fasting reduced LC3-positive dots in both WT and UVRAG-deficient mice during the feeding day 5 days after initial DOX challenge (Fig. 6a,b). Alteration of LC3 II and p62 protein abundance was in parallel with LC3-postive dots (Fig. 6e–g). In chronic DOX-induced cardiotoxicity, LC3-postive dots were also reduced by intermittent fasting (Fig. 6c,d). In addition, LC3 II and p62 protein levels in LVs from DOX-treated WT and UVRAG-deficient mice were significantly decreased by intermittent fasting (Fig. 6h–j). These data suggest that intermittent fasting rescues autophagy in the hearts from DOX-treated WT and UVRAG-deficient mice.

Interestingly, intermittent fasting reduced lysosomal markers LAMP-1 and LAMP-2 in LVs from WT mice after acute but not chronic DOX treatment (Figs S7a,b and S8a,b). Moreover, intermittent fasting had no effect on LAMP-1 and LMAP-2 pattern in LVs from UVRAG-deficient mice after acute and chronic DOX treatment (Figs S7a,b and S8a,b), indicating that chronic DOX treatment and DOX treatment combining UVRAG deficiency cause more severe damage to lysosomes or autolysosomes in the heart.

### Intermittent fasting ameliorates pathology of DOX-induced cardiotoxicity

**Figure 7:**
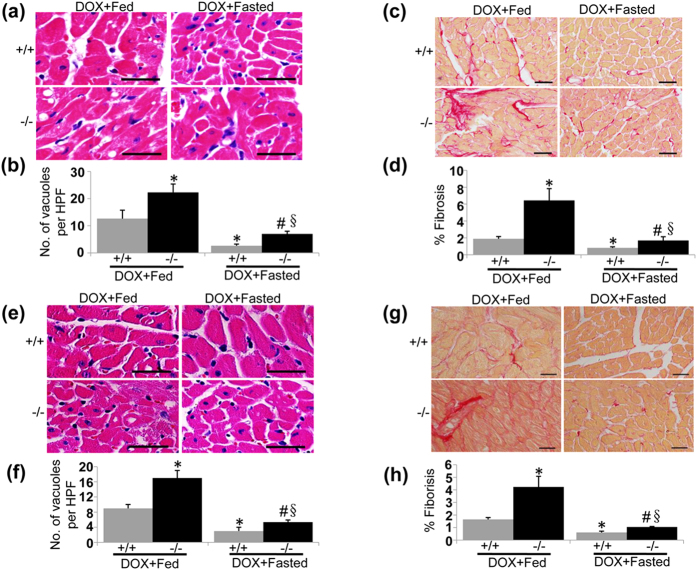
Intermittent fasting ameliorates pathology of DOX-induced cardiotoxicity. (**a**) Representative H&E images of degenerative vacuoles in LVs on heart sections from fed or fasted WT and UVRAG-deficient mice 5 days after acute DOX or vehicle treatment. Scale bar: 40 μm. (**b**) Quantification of degenerative vacuoles in LVs in the experiments as illustrated in (**a**). n = 3 mice for each group. (**c**) Representative images of fibrosis stained with picrosirius red in LVs on heart sections from fed or fasted WT and UVRAG-deficient mice 5 days after acute DOX or vehicle treatment. Scale bar: 40 μm. (**d**) Quantification of fibrosis in LVs in the experiments as illustrated in (**c**). n = 3 mice for each group. (**e**) Representative H&E images of LVs on heart sections from fed or fasted WT and UVRAG-deficient mice at 4 weeks of DOX treatment in chronic cardiotoxicity. Scale bar: 40 μm. (**f**) Quantification of degenerative vacuoles in LVs in the experiments as illustrated in (**e**). n = 3 mice for each group. (**g**) Representative images of fibrosis stained with picrosirius red in LVs on heart sections from fed or fasted WT and UVRAG-deficient mice at 4 weeks of DOX treatment in chronic cardiotoxicity. (**h**) Quantification of fibrosis in LVs in the experiments as illustrated in (**g**). n = 3 mice for each group. Scale bar: 40 μm. *P < 0.05 vs. WT + DOX + Fed, ^#^*P* < 0.05 vs. UVRAG^−/−^ + DOX + Fed, ^§^*P* < 0.05 vs. WT + DOX + Fasted.

**Figure 8:**
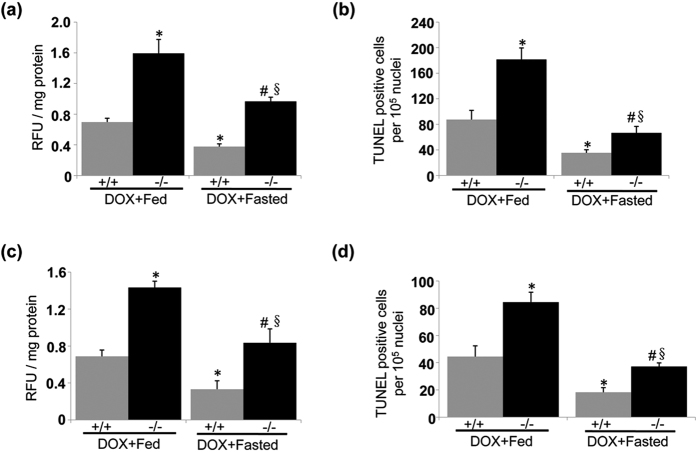
Intermittent fasting attenuates enhanced ROS levels and increased apoptotic cell death in UVRAG-deficient mice treated by DOX. (**a**) Intermittent fasting rescued enhanced ROS levels in LVs from UVRAG-deficient mice measured 5 days after acute DOX treatment. n = 3 mice for each group. (**b**) Intermittent fasting attenuated increased apoptotic cell death in the hearts from UVRAG-deficient mice assessed 5 days after acute DOX treatment. n = 3 mice for each group. (**c**) Intermittent fasting rescued enhanced ROS levels in LVs from UVRAG-deficient mice at 4 weeks of DOX treatment in chronic DOX cardiotoxicity. n = 3 mice for each group. (**d**) Intermittent fasting attenuated increased apoptotic cell death in the hearts from UVRAG-deficient mice at 4 weeks of DOX treatment in chronic cardiotoxicity. n = 3 mice for each group. *P < 0.05 vs. WT + DOX + Fed, ^#^*P* < 0.05 vs. UVRAG^−/−^ + DOX + Fed, ^§^*P* < 0.05 vs. WT + DOX + Fasted.

Our present results suggest that impaired autophagic flux contributes to the pathogenesis of DOX-induced cardiotoxicity. Given that intermittent fasting is capable of restoring autophagy in this scenario, therefore, we propose that intermittent fasting ameliorates pathology of DOX-induced cardiotoxicity. Intermittent fasting mitigated degenerative vacuoles and fibrosis in LVs from WT and UVRAG-deficient mice after acute (Fig. 7a–d) and chronic (Fig. 7e–h) DOX treatment. Moreover, intermittent fasting attenuated enhanced ROS levels and increased apoptosis in the hearts from WT and UVRAG-deficient mice following acute (Fig. 8a,b) and chronic (Fig. 8c,d) DOX treatment. Thus, intermittent fasting mitigates pathology of DOX-induced cardiotoxicity in both WT and UVRAG-deficient mice.

## Discussion

Autophagy has recently been linked to DOX-induced cardiomyopathy. Previously we have shown that UVRAG regulates autophagic flux in the heart at autophagosome maturation step^23^. Here we sought to determine the role of UVRAG-mediated autophagy in DOX-induced cardiotoxicity. To accomplish this, we established mouse models of acute and chronic DOX-induced cardiotoxicity frequently used mimicking acute and chronic DOX cardiotoxicity in patients^42^,^43^,^44^. A major finding of this study was that UVRAG deficiency exacerbated DOX-induced cardiotoxicity. In addition, the survival rate was significantly lower in UVRAG-deficient mice compared with WT controls after DOX treatment. We cannot attribute the mortality to cardiotoxicity since DOX induces systemic damage. However, DOX-treated UVRAG-deficient mice did indeed show more severe cardiac injury manifested by increased degenerative vacuoles, pronounced collagen accumulation, elevated serum LDH and CK-MB activities, higher ROS levels, aggravated apoptosis and enhanced inflammation.

ROS has been considered the major mediator in DOX-induced cardiotoxicity. Mitochondria, which are abundant in cardiomyocyte, are the main source of ROS. Turnover of damaged mitochondria through autophagy is essential to maintain cardiomyocyte structure and function^33^. Therefore, enhanced levels of ROS in LVs from DOX-treated UVRAG-deficient mice are at least partially attributed to more severe impairment of autophagic flux. Iron accumulation in the mitochondria has recently been shown to cause DOX cardiotoxicity largely through promoting ROS generation^7^. Future research is needed to investigate regulation of mitochondrial iron accumulation by UVRAG in DOX cardiotoxicity. We cannot rule out the possibility that defects other than autophagy resulting from UVRAG deficiency contribute to exacerbated DOX-induced cardiotoxicity.

Inflammatory cytokines are elevated by UVRAG deficiency after DOX treatment. Previous studies show that ROS is capable of inducing expression of inflammatory cytokines such as TNFα, which in turn promotes ROS generation, leading to a vicious cycle^45^. In addition, mitochondrial DNA that escapes from autophagic degradation causes inflammation and cardiac dysfunction^46^. UVRAG deficiency aggravates DOX-induced impaired autophagic flux, which may increase free mitochondrial DNA in cardiomyocytes and promote inflammation.

The key question is what are the potential mechanisms that DOX impairs autophagic flux and UVRAG deficiency aggravates impaired autophagic flux in DOX-induced cardiotoxicity? By means of detecting LC3 II and p62 protein abundance in the heart elicited by CQ-treated WT mice, we showed that autophagic flux was impaired in acute and chronic DOX-induced cardiotoxicity, which is in agreement with several other studies^15^,^47^. Interestingly, LC3 II and p62 in LVs from DOX-treated animals were more abundant compared with animals treated with CQ alone. CQ was reported to inhibit autophagic flux by altering lysosomal PH^48^, which was shown somehow elevated in DOX-treated cardiomyocyte^47^ while this manuscript was in preparation. Therefore, CQ and DOX exert overlapping functions at this point. In addition, DOX increases autophagic activation while blunting autophagosome formation^41^. We also showed that Beclin 1, a positive mediator of autophagy induction, was upregulated in DOX-treated animals. Thus, the enhanced LC3 II in DOX-treated mice compared with mice treated with CQ alone may be caused by combinatorial effects of DOX on increased autophagic induction, impaired autophagosome formation and lysosomal degradation. Previously, autophagosome maturation has been suggested to be impaired in DOX-induced cardiomyopathy^15^. Our recent studies have shown that UVRAG deficiency impairs autophagic flux in the heart through inhibiting autophagosome maturation. Here, we showed that LC3 II and p62 protein abundance were enhanced in the hearts from UVRAG-deficient mice compared with WT controls after DOX treatment, suggesting UVRAG deficiency further blocks autophagosome maturation and subsequent lysosomal degradation. Thus, autophagosome maturation is blunted but not completely suppressed in DOX-induced cardiotoxicity. Taken together, our results and previous work suggest that multiple crucial steps of autophagy including autophagosome formation, maturation and autolysosomal degradation are blunted in DOX-induced cardiotoxicity, resulting in reduced autophagic flux.

Fasting exerts a number of beneficial effects on health mediated by remodeling of mitochondria dynamics, enhanced mitochondria biogenesis, changes in energy metabolism, decreased signaling pathway associated with survival such as insulin and insulin-like growth factor-1 signaling, reduction in mitochondria oxidative stress and promotion of autophagy^37^. Fasting has been demonstrated to activate autophagy regulator TFEB, which in turn promotes expression of genes involved in sequential stages of autophagy, leading to enhanced autophagic protein degradation^49^. Given that several crucial steps of autophagic pathway were targeted by DOX in the heart, it is reasonably expected that fasting could be efficient to restore autophagic flux in DOX-induced cardiotoxicity. Intermittent fasting is capable of boosting cardiac autophagy in a cyclic manner under normal conditions^40^ and meanwhile avoids adverse effects generated by prolonged fasting. Therefore, we tested the effects of intermittent fasting on autophagy in acute and chronic DOX-induced cardiotoxicity. We provided evidence that intermittent fasting reduced the accumulation of autophagic vacuoles and decreased protein levels of LC3 II and p62, suggesting that autophagy is restored in DOX-induced cardiotoxicity. However, the precise mechanisms remain to be elucidated. In addition to restoring autophagy, intermittent fasting ameliorated pathology of DOX-induced cardiotoxicity in WT and UVRAG-deficient mice manifested by reduced degenerative vacuoles, collagen accumulation, ROS levels and apoptosis, indicating that impaired autophagy contributes to DOX-induced cardiotoxicity.

Interestingly, large LAMP-1- and LAMP-2-positive dots examined by immunohistochemistry were present in the hearts following DOX treatment. More importantly, intermittent fasting reduced LAMP-1- and LAMP-2-positive dots in the hearts following acute but not chronic DOX treatment. Thus, chronic DOX treatment may cause more severe damage to lysosomes or autolysosomes. Furthermore, intermittent fasting showed no impact on LAMP-1 or LAMP-2-positive dots in DOX-treated UVRAG-deficient mice, consistent with the fact that UVRAG regulates ALR^22^.

In conclusion, UVRAG deficiency leads to exacerbation of DOX-induced cardiotoxicity, at least in part, through aggravation of impaired autophagic flux. Intermittent fasting is capable of rescuing impaired autophagy and ameliorating pathology of DOX-induced cardiotoxicity. Given that intermittent fasting and multiple fasting cycles suppress tumor growth and sensitize a wide range of tumors to chemotherapy^50^,^51^, intermittent fasting could be considered as a potential preventive or therapeutic strategy for cardiotoxicity induced by DOX in the treatment of cancer. However, the fasting protocol needs to be optimized to improve efficacy while minimizing adverse effects.

## Materials and Methods

### Experimental animals

All mice used in this study were of the FVB/NJ background. Animal procedures were conducted in accordance with the Guide for the Care and Use of Laboratory Animals (Directive 2010/63/EU of the European Parliament), with the experimental protocols approved by the Institutional Animal Care and Use Committee of Shanghai, China [SYXK(SH)2011-0112]. Generation of UVRAG-deficient mice was described previously^23^,^24^.

Acute DOX-induced cardiotoxicity was induced in 2-month-old WT and UVRAG-deficient mice by a single intraperitoneal injection of DOX (20 mg/kg, BBI, Toronto, Canada) dissolved in saline. Chronic DOX-induced cardiotoxicity was induced in eight-week-old WT and UVRAG-deficient mice by intraperitoneally injected 6 doses of DOX (4 mg/kg, dissolved in saline) at weekly intervals. Mice were observed for another 2 weeks before assessment of cardiac function.

In both acute and chronic DOX cardiotoxicity, mice were anaesthetized by isoflurane inhalation and euthanized by rapid cervical dislocation at the indicated time points and left ventricles were dissected from the excised hearts for western blot and ROS analysis. For histological and immunohistochemical staining, mice were anaesthetized by isoflurane inhalation and the entire hearts were used to prepare paraffin-embedded serial sections in four-chamber view. Generally time-course analysis of vacuolar degeneration and fibrosis in LVs were performed in acute and chronic DOX cardiotoxicity. ROS and apoptosis were assessed 5 days after DOX treatment in acute DOX-induced cardiotoxicity and at 4 weeks of DOX treatment in chronic DOX-induced cardiotoxicity.

In the fasting experiments, mice were subjected to intermittent (alternate-day) fasting, starting from one day before the initial DOX treatment. Fasted mice had free access to water. LVs or the perfusion-fixed hearts from the fasted group were dissected during the day of feeding after fasting treatment as indicated. In this study, we dissect left ventricle to make consistent tissue sampling to exclude the possible impacts of different hemodynamics between left ventricle and right ventricle on the experimental results.

### Antibodies

Mouse anti-LC3 monoclonal antibody (M152-3), Rabbit anti-LC3 polyclonal antibody (PM036) and rabbit anti-Beclin 1 polyclonal antibody (PD017) were from MBL International Corporation (Nagoya, Japan), rabbit anti-p62 polyclonal antibody (#5114), rabbit anti-Atg5 monoclonal antibody (#12994) and rabbit anti-UVRAG monoclonal antibody (#13115) were purchased from Cell Signaling Technology (Danvers, MA, USA), mouse anti-tubulin monoclonal antibody (628802) was obtained from Gene Tex, Inc (Irvine, CA, USA), rat anti-LAMP-1 monoclonal antibody (sc19992), rat anti-LAMP-2 monoclonal antibody (sc19991), mouse anti-ubiquitin monoclonal antibody (sc8017), rabbit anti-XIAP polyclonal antibody (sc5550) and rabbit anti-GAPDH polyclonal antibody (sc25778) were from Santa Cruz Biotechnology (Dallas, TX, USA), rabbit anti-α-smooth muscle actin (α-SMA) polyclonal antibody was obtained from Abcam Limited (Cambs, UK), and mouse anti-β-actin monoclonal antibody (AM1021B) was purchased from ABGENT (Suzhou, China).

### Histology and immunohistochemistry

Following anesthesia, mouse hearts were perfused with ice cold PBS followed by fixation by 4% paraformaldehyde in PBS. Fixed hearts were embedded in paraffin and cut into serial sections in four-chamber view at 5 μm in a Leica RM2255 rotary microtome (Leica Microsystems, Wetzlar, Germany). Heart sections were stained with H&E or picrosirius red for evaluation of routine morphology and collagen accumulation. Immunohistochemistry was performed on the paraffin sections using the VECTASTAIN^®^ Elite ABC Kit (Vector Laboratories, Burlingame, CA, USA) as previously described^23^. TUNEL assay was performed on the paraffin sections using an ApopTag^®^ Plus Peroxidase *In Situ* Apoptosis Detection Kit (Millipore, MA, USA) according to the manufacturer’s instructions. For quantification of vacuolar degeneration of cardiomyocytes, fibrosis and α-SMA expression, around 20 microscopic fields randomly selected in LVs per section, 3 sections from each of 3–5 mice were assessed. For quantification of apoptotic cells in the heart, 20–25 microscopic fields randomly selected per section, 3 sections from each of 3 mice were evaluated by TUNEL assay.

### Measurement of LDH and CK-MB

Serum LDH activity was assayed by means of reflectance spectroscopy using LDH detection kit (Nanjing Jiancheng Bioengineering Institute, Nanjing, China). Serum CK-MB activity was determined using mouse CK-MB ELISA kit (Nanjing Jiancheng Bioengineering Institute, Nanjing, China). Determination of LDH and CK-MB were performed according to the manufacturer’s instructions.

### Autophagic flux assessment

*In vivo* autophagic flux assessment was evaluated as described previously^23^. In brief, mice were intraperitoneally injected with vehicle (ddH_2_O) or chloroquine (CQ, 50 mg/kg, Sigma-Aldrich, St. Louis, MO, USA) and LVs from excised hearts were harvested for analysis of LC3 II and p62 protein levels by western blot. Immunohistochemical staining for LC3 II was performed on paraffin-embedded heart sections.

### ROS measurement

Analysis of ROS was performed as described previously^52^. Briefly, 10 mg of fresh left ventricular tissue was homogenized in 1 ml of ice cold 40 mM Tris–HCl buffer (pH 7.4) and the homogenate was diluted to 0.25% with the same buffer. Samples were then loaded with 40 μL 1.25 mM 2′,7′-dichlorofluorescin-diacetate (DCFH-DA, Sigma-Aldrich, St. Louis, MO, USA) at 37 °C for 30 min. The fluorescence intensity was measured by a fluorescent microplate reader with excitation wave length at 488 nm and emission wave length at 535 nm. Results were expressed as relative fluorescence unit (RFU) per mg protein.

### Assessment of inflammatory cytokines

TNFα, IL-6 and IL-1β were assessed in heart homogenates using ELISA kits (Nanjing Jiancheng Bioengineering Institute, Nanjing, China) according to the manufacturer’s instructions.

### Western blot

Proteins extracted from left ventricular tissues were separated by SDS-PAGE gel and transferred to nitrocellulose membrane. The membranes were then blocked for 1 hour at room temperature with 5% skim milk powder in Tris-buffered saline with Tween 20 (TBST) buffer and incubated with primary antibodies overnight at 4 °C. After washing with TBST buffer, the membranes were incubated with secondary antibodies conjugated to horseradish peroxidase for 1 hour at room temperature and again washed with TBST buffer. The protein bands were detected by using Immobilon^TM^ Western Chemiluminescent HRP Substrate (Millipore, MA, USA). All western blot quantifications were performed using the Image J software.

### Echocardiography

Echocardiography was performed as described previously^53^. Briefly, mouse chests were shaved one day before the measurement. Mice were placed on a heating plate for anesthesia by inhaled isoflurane. Echocardiographic imaging was conducted using a Vevo 770 platform (VisualSonics Inc., Toronto, ON, CAN) equipped with a 45 MHz imaging transducer. Measurements were carried out at least in triplicate.

### Statistics

All data were expressed as mean ± S.E.M. Statistical differences were analyzed by one-way ANOVA followed by Bonferroni’s test for multiple comparisons using GraphPadPrism software. Values of *P* < 0.05 were considered statistically significant.

## Additional Information

**How to cite this article**: An, L. *et al*. UVRAG Deficiency Exacerbates Doxorubicin-Induced Cardiotoxicity. *Sci. Rep.* **7**, 43251; doi: 10.1038/srep43251 (2017).

**Publisher's note:** Springer Nature remains neutral with regard to jurisdictional claims in published maps and institutional affiliations.

## Supplementary Material

Supplementary Information
