# Supplementary material for: UVRAG Deficiency Exacerbates Doxorubicin-Induced Cardiotoxicity
Source: Sci Rep. 2017 Feb 22;7:43251. doi: 10.1038/srep43251 (PMC5320807; doi:10.1038/srep43251)
Supplement: Supplementary Information [file srep43251-s1.pdf]

Supporting information

UVRAG Deficiency Exacerbates Doxorubicin-Induced Cardiotoxicity

Lin An[1], Xiao-wen Hu[1], Shasha Zhang[1], Xiaowen Hu[1], Zongpei Song[1], Amber Naz[1], Zhenguo Zi[1], Jian Wu[2], Can Li[1,3], Yunzeng Zou[2], Lin He[1,3], Hongxin Zhu[1,3]

1 *Bio-X-Renji Hospital Research Center, Renji Hospital, School of Medicine, Shanghai Jiao Tong University*

2 *Institutes of Biomedical Sciences, Fudan University, Shanghai, China*

3 *Collaborative Innovation Center of Genetics and Development, China*

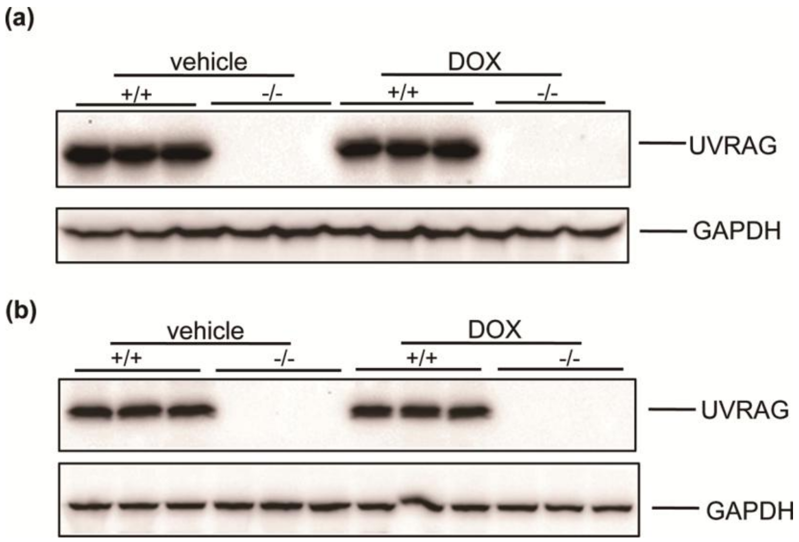

Figure S1. UVRAG expression in the hearts from WT and UVRAG-deficient mice after vehicle and DOX treatment. (a) Western blot detection of UVRAG protein abundance in LVs from WT and UVRAG-deficient mice 5 days after acute DOX or vehicle treatment. **(b)** Western blot detection of UVRAG protein abundance in LVs from WT and UVRAG-deficient mice at 4 weeks of chronic DOX or vehicle treatment.

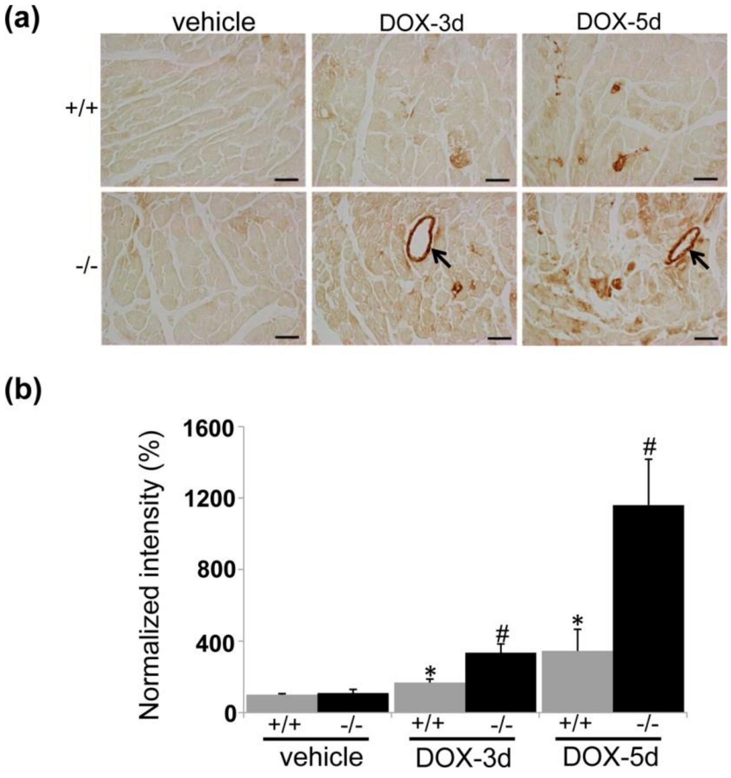

Figure S2. Expression of α-SMA in the hearts from WT and UVRAG-deficient mice after acute DOX and vehicle treatment. (a) Representative images of α-SMA immunohistochemistry in LVs on heart sections from WT and UVRAG-deficient mice after chronic DOX or vehicle treatment over the indicated time. Scale bar: 40 μm. **(b)** Quantification of α-SMA expression in LVs in the experiments as illustrated in (a). n=3 mice for each group. *P＜0.05 vs. WT+vehicle, #P＜0.05 vs. WT+DOX.

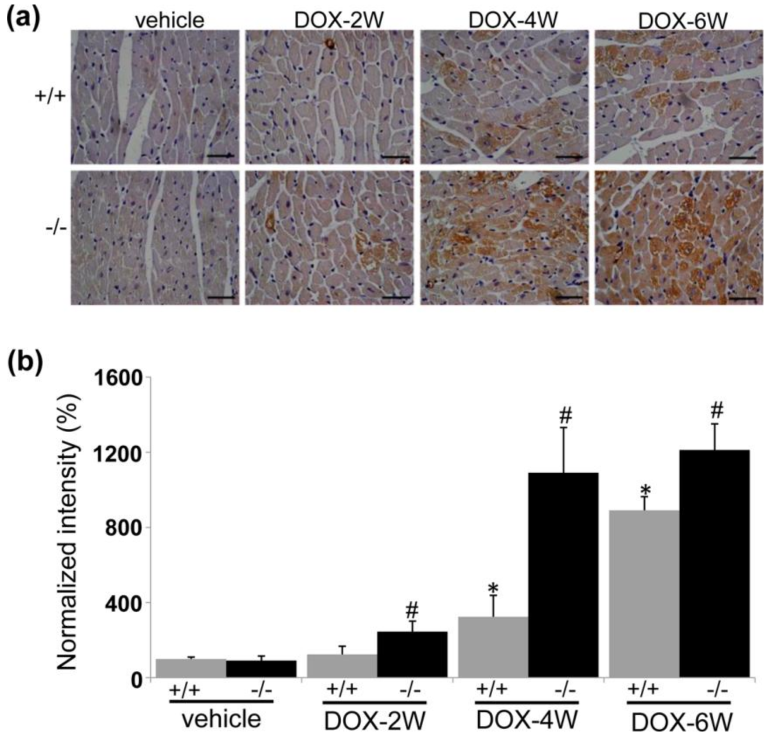

Figure S3. Expression of α-SMA in the hearts from WT and UVRAG-deficient mice after chronic DOX and vehicle treatment. (a) Representative images of α-SMA immunohistochemistry in LVs on heart sections from WT and UVRAG-deficient mice after chronic DOX or vehicle treatment over the indicated time. Scale bar: 40 μm. **(b)** Quantification of α-SMA expression in LVs in the experiments as illustrated in panel (a). n=3 mice for each group. *P＜0.05 vs. WT+vehicle, #P＜0.05 vs. WT+DOX.

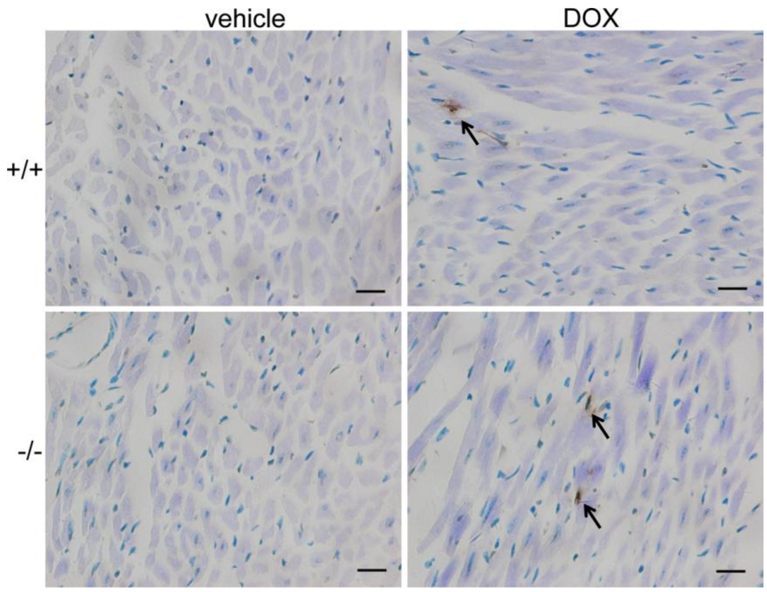

Figure S4. Representative images of apoptotic cells detected by TUNEL assay. Scale bar: 40 μm.

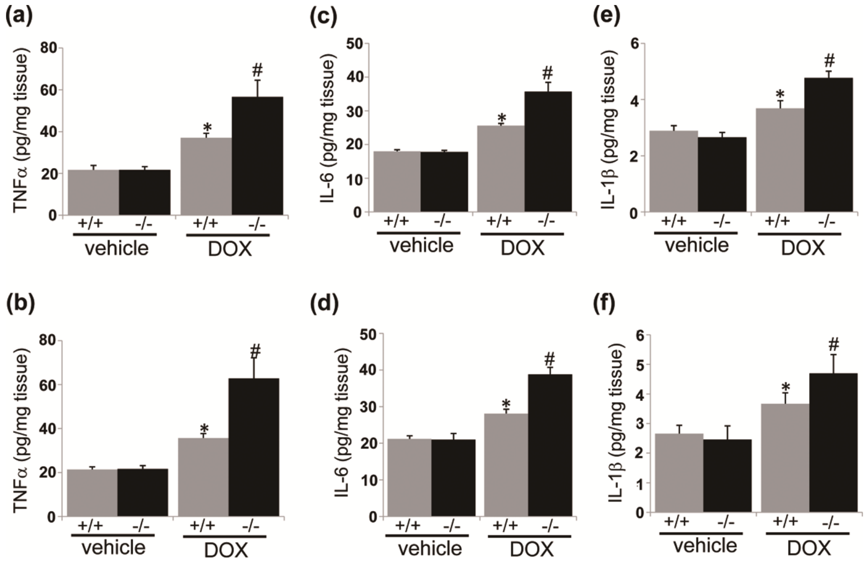

Figure S5. Assessment of inflammatory cytokines in acute and chronic DOX-induced cardiotoxicity. **(a)** TNFα levels in the hearts from WT and UVRAG-deficient mice 3 days after vehicle or DOX treatment. **(b)** TNFα levels in the hearts from WT and UVRAG-deficient mice at 2 weeks of chronic DOX or vehicle treatment. **(c)** IL-6 levels in the hearts from WT and UVRAG-deficient mice 3 days after vehicle or DOX treatment. **(d)**

IL-6 levels in the hearts from WT and UVRAG-deficient mice at 2 weeks of chronic DOX or vehicle treatment. **(e)** IL-1β levels in the hearts from WT and UVRAG-deficient mice 3 days after vehicle or DOX treatment. **(f)** IL-1β levels in the hearts from WT and UVRAG-deficient mice at 2 weeks of chronic DOX or vehicle treatment. n=4 mice for each group. *$P < 0.05$ vs. WT+vehicle. # $P < 0.05$ vs. WT+DOX.

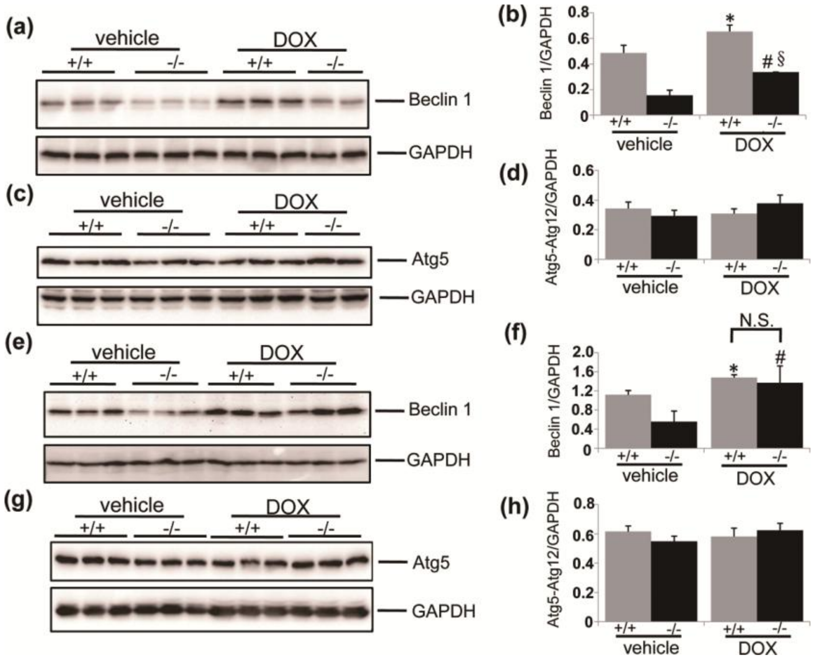

Figure S6. Beclin 1 and Atg5 expression in the hearts from WT and UVRAG-deficient mice after vehicle and DOX treatment. (a) Western blot detection of Beclin 1 protein abundance in LVs from WT and UVRAG-deficient mice 3 days after acute DOX or vehicle treatment. **(b)** Quantification of Beclin 1 as illustrated in panel (a). **(c)** Western blot detection of Atg5 protein abundance in LVs from WT and UVRAG-deficient mice 3 days after acute DOX or vehicle treatment. **(d)** Quantification of Atg5 as illustrated in panel (c). **(e)** Western blot detection of Beclin 1 protein abundance in LVs from WT and UVRAG-deficient mice at 4 weeks of chronic DOX or vehicle treatment. **(f)** Quantification

of Beclin 1 as illustrated in panel (e). **(g)** Western blot detection of Atg5 protein abundance in LVs from WT and UVRAG-deficient mice at 4 weeks of chronic DOX or vehicle treatment. **(h)** Quantification of Atg5 as illustrated in panel (g). **P*＜0.05 vs. WT+vehicle, #*P*＜0.05 vs. UVRAG$^{-/-}$+vehicle，§*P*＜0.05 vs. WT+DOX.

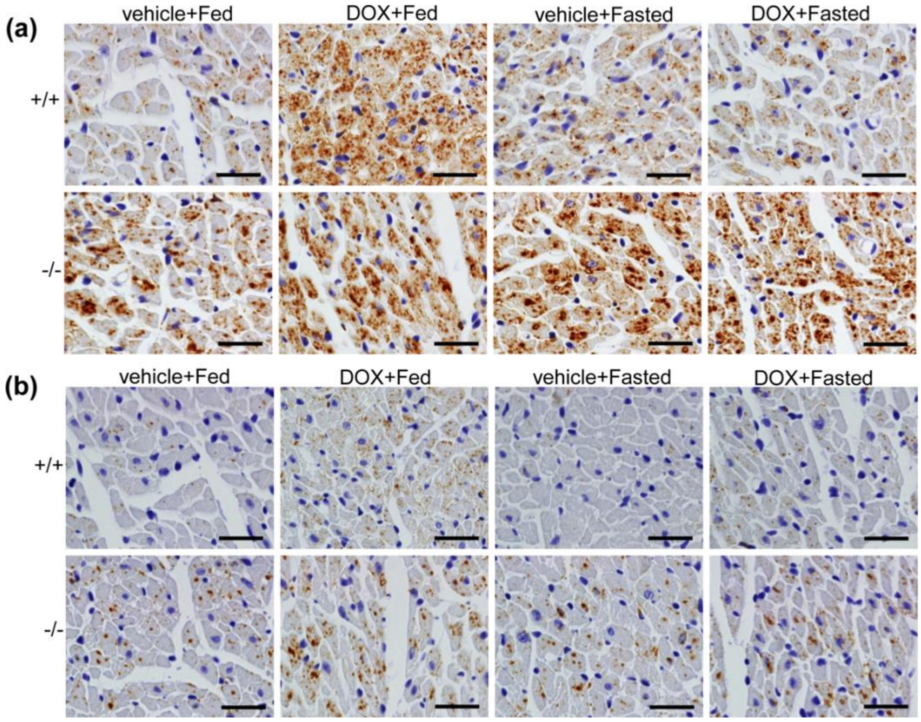

Figure S7. Effects of intermittent fasting on lysosomal markers in acute cardiotoxicity. **(a)** Representative images of immunohistochemistry for LAMP-1 in LVs on the heart sections from fed or fasted WT and UVRAG-deficient mice 5 days after acute DOX or vehicle treatment. **(b)** Representative images of immunohistochemistry for LAMP-2 in LVs on the heart sections from fed or fasted WT and UVRAG-deficient mice 5 days after acute DOX or vehicle treatment. Scale bar: 40 μm.

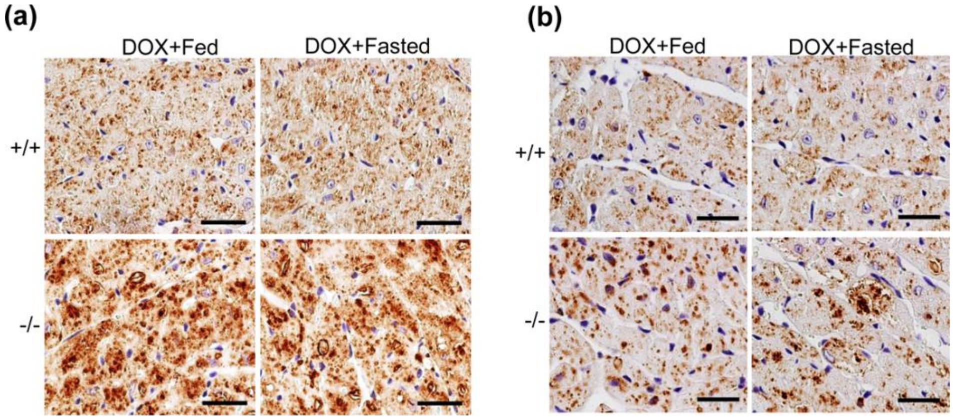

Figure S8. Effects of intermittent fasting on lysosomal markers in chronic cardiotoxicity. **(a)** Representative images of immunohistochemistry for LAMP-1 in LVs on the heart sections from fed or fasted WT and UVRAG-deficient mice 4 weeks after the initial DOX treatment in chronic cardiotoxicity. **(b)** Representative images of immunohistochemistry for LAMP-2 in LVs on the heart sections from fed or fasted WT and UVRAG-deficient mice 4 weeks after the initial DOX treatment in chronic cardiotoxicity. Scale bar: 40 μm.
